# Hypercoagulability and Risk of Venous Thromboembolic Events in Endogenous Cushing's Syndrome: A Systematic Meta-Analysis

**DOI:** 10.3389/fendo.2018.00805

**Published:** 2019-01-28

**Authors:** Jeffrey Wagner, Fabienne Langlois, Dawn Shao Ting Lim, Shirley McCartney, Maria Fleseriu

**Affiliations:** ^1^Northwest Pituitary Center, Oregon Health & Science University, Portland, OR, United States; ^2^Centre Hospitalier Universitaire de Sherbrooke, Fleurimont, QC, Canada; ^3^Singapore General Hospital, Singapore, Singapore

**Keywords:** venous thromboembolism, Cushing disease, Cushing syndrome, hypercoagulability, anticoagulation

## Abstract

**Background:** Hypercortisolism has been implicated in the development of venous thromboembolic events (VTE). We aimed to characterize VTE risk in endogenous Cushing's syndrome (CS) patients, compare that risk to other pathologies, and determine if there are any associated coagulation factor changes.

**Methods:** Medline and Scopus search for “hypercortisolism” and “thromboembolic disease” from January 1980 to April 2017 to include studies that reported VTE rates and/or coagulation profile of CS patients. A systematic review and meta-analysis were performed.

**Results:** Forty-eight studies met inclusion criteria. There were 7,142 CS patients, average age was 42 years and 77.7% female. Odds ratio of spontaneous VTE in CS is 17.82 (95%CI 15.24–20.85, *p* < 0.00001) when comparing to a healthy population. For CS patients undergoing surgery, the odds ratio (both with / without anticoagulation) of spontaneous VTE is 0.26 (95%CI 0.07–0.11, *p* < 0.00001)/0.34 (0.19–0.36, *p* < 0.00001) when compared to patients undergoing hip fracture surgery who were not treated with anticoagulants. Coagulation profiles in patients with CS showed statistically significant differences compared to controls, as reflected by increases in von Willebrand factor (180.11 vs. 112.53 IU/dL, *p* < 0.01), as well as decreases in activated partial thromboplastin time (aPTT; 26.91 vs. 30.65, *p* < 0.001) and increases in factor VIII (169 vs. 137 IU/dL, *p* < 0.05).

**Conclusion:** CS is associated with significantly increased VTE odds vs. general population, but lower than in patients undergoing major orthopedic surgery. Although exact timing, type, and dose of anticoagulation medication remains to be established, clinicians might consider monitoring vWF, PTT, and factor VIII when evaluating CS patients and balance advantages of thromboprophylaxis with risk of bleeding.

## Introduction

Endogenous Cushing's syndrome (CS) results in multiple systemic manifestations related to excess cortisol, including catabolic effects, central fat redistribution and psychiatric manifestations (1). Endogenous CS can be stratified into two subtypes; adrenocorticotropic hormone (ACTH)-dependent CS, where excess ACTH leads to overproduction of cortisol, or ACTH-independent disease where cortisol is produced regardless of input from ACTH. The former is attributed to an ACTH-secreting pituitary adenoma (Cushing's disease; CD), or ectopic ACTH-secretion while autonomous adrenal hypersecretion of cortisol results in ACTH-independent disease (2–4). The most common is ACTH-dependent CS, where an estimated 70% of cases are due to a pituitary adenoma; CD (5). Though probably underdiagnosed, reported incidence of CD is between 6.2 and 7.6 per million patient years, while the reported incidence of adrenal CS is approximately 0.6 per million per year (3, 4). First-line treatment in most cases is surgical resection of the culprit tumor; some patients may further require medical therapy, bilateral adrenalectomy, and/or radiation (4, 6).

An association between hypercortisolism and hypercoagulability has been previously documented (7–11). A state of hypercoagulability reflects German pathologist Rudolph Virchow's postulation that venous thrombosis and/or arterial thrombosis are preceded by identifiable factors (12). Risk of postoperative venous thromboembolism events (VTE) was significantly higher in patients with CD compared to patients undergoing pituitary surgery for a non-functioning pituitary adenoma, suggesting that the high risk for postoperative VTE in CS is cortisol mediated (13). Importantly, other disease states, such as patients undergoing orthopedic surgery for hip replacement, are routinely treated with thromboprophylaxis (14). However, there is no consensus about the exact risk of thrombosis in patients with endogenous CS. Quantifying this risk is important to balance the advantages and complications associated with anticoagulation treatment. The Endocrine Society Guidelines for Treatment of Cushing's syndrome suggests clinicians be aware of the altered coagulation profile of CS patients for up to 1-year following surgical cure, and consider anticoagulation on an individual basis (15). The guidelines, however, do not offer screening metrics for venous thromboembolism (VTE) assessment risk in CS patients. Further, few observational studies and case-series have demonstrated changes in von Willebrand factor (vWF), factor-VIII (fVIII), or homocysteine in CS patients (9, 16–19). Other coagulation markers could also play a role (20–22).

Here, we performed a meta-analysis to study the association between endogenous hypercortisolism and hypercoagulability (venous thrombosis events and alteration of coagulation profile). We hypothesized that patients with hypercortisolism due to CS have a significantly increased risk of VTE compared to the general population. We also reviewed available evidence of coagulation profiles for CS patients and compared to the general population. Lastly, we quantified the odds of VTE in CS and compared it with the odds of patients undergoing major orthopedic surgery.

## Materials and Methods

### Study Identification

Studies were identified using search terms in both MEDLINE and SCOPUS databases up to April 2017. The query in PubMed, the search engine to access the MEDLINE database, included MeSH terms and non-MeSH. Terms were related to both endogenous hypercortisolism and hypercoagulability, more specifically detailed in Table 1.

**Table 1:** Search terms used as of April, 2017.

**Database search**	**Terms**
MEDLINE	1. (((((((((“Venous Thromboembolism”[Mesh]) OR “Venous Thrombosis”[Mesh]) OR “Pulmonary Embolism”[Mesh]) OR “Thrombophilia”[Mesh]) OR “Hemostasis”[Mesh]) OR “Blood Coagulation”[Mesh]) OR “Thromboembolism”[Mesh]) OR “Pulmonary Infarction”[Mesh])) AND ((((((“Cushing Syndrome”[Mesh]) OR “Pituitary ACTH Hypersecretion”[Mesh]) OR “Acth-Independent Macronodular Adrenal Hyperplasia” [Supplementary Concept]) OR “ACTH-Secreting Pituitary Adenoma”[Mesh]) OR “ACTH Syndrome, Ectopic”[Mesh]) OR “Glucocorticoids”[Mesh]) 803 results of all MeSH terms
	2. (((((((((((((hemostatic) OR blood coagulation) OR hypercoagulability) OR prothrombotic state) OR thrombosis) OR thromboembolism) OR venous thromboembolism) OR deep vein thrombosis) OR pulmonary embolism) OR pulmonary infarction) OR embolus) OR clot)) AND (((((((((((adrenocortical hyperplasia) OR cushing disease) OR cushing syndrome) OR hypercortisolism) OR adrenocortical hyperfunction) OR adrenocortical adenoma) OR corticotropin adenoma) OR acth secreting pituitary adenoma) OR inappropriate acth secretion syndrome) OR pituitary acth hypersecretion) OR pituitary hyperplasia) 724 results of all field search
SCOPUS	(TITLE-ABS-KEY (cushing's OR “cushing's disease” OR “cushing's syndrome” OR glucocorticoid OR “ectopic acth syndrome” OR hypercortisolism) AND TITLE-ABS-KEY (“venous thromboembolism” OR “venous thrombosis” OR “pulmonary embolism” OR thrombophilia OR “blood coagulation” OR thromboembolism OR hypercoagulability OR “prothrombotic state”)) 1,127 results of all field search

**Figure 1:**
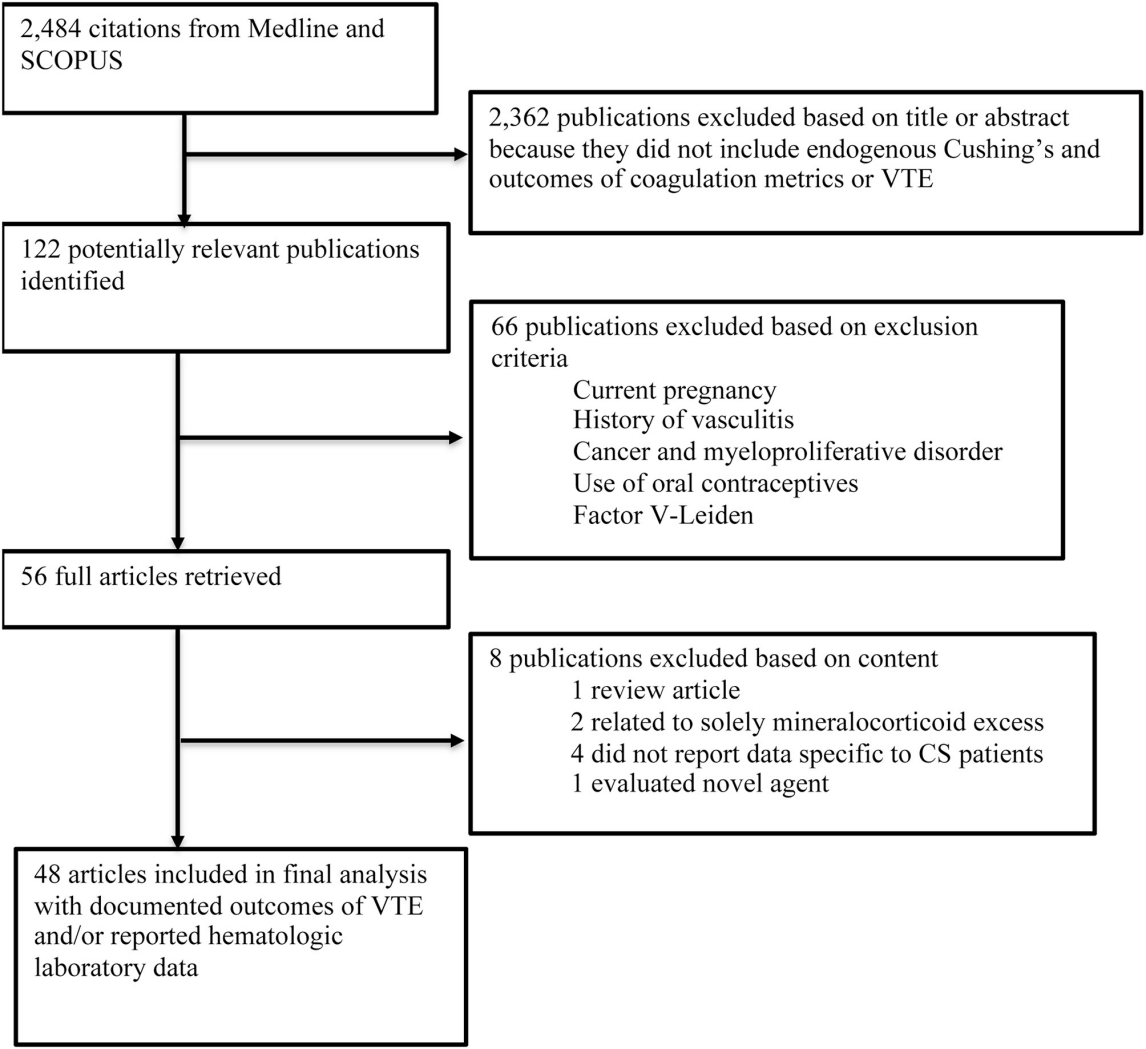
Flow chart of the search strategy and selection.

In MEDLINE, a cumulative search generated 803 articles using MeSH terms and 724 articles using non-MeSH terms. The same search terms were used in SCOPUS, which identified 1,127 articles. References from included articles were also searched for additional articles. Articles from both databases queries were combined to compromise comprehensive review of the literature. Duplicate studies identified in both databases were reduced so each article was only accounted for once in the cumulative dataset with all duplicates removed, resulting in 2,484 articles (Figure 1).

The titles and abstracts were reviewed by two of three independent reviewers. Inclusion criteria included both diagnosis of hypercortisolism due to endogenous CS and either reporting of VTE events or coagulation laboratory data. The search was also limited to human studies published after 1980 in English-language literature, and reports of more than 1 patient. Studies that included patients undergoing treatment with systemic supraphysiological glucocorticoids were excluded. Similarly, malignant causes of CS, mostly ectopic ACTH-syndromes were also excluded. Notable exceptions were made for studies that pooled total of 81 CS patients with ectopic ACTH in their analyses where authors did not stratify patients by underlying diagnosis. Those studies were not excluded since included the majority of patients had hypercortisolism of pituitary or adrenal origin.

Exclusion criteria also included studies that were unrelated to CS population or any of the following: diagnosis of current pregnancy, vasculitis, cancer or myeloproliferative disease, systemic lupus erythematosus or other inflammatory condition, heparin induced thrombocytopenia, antithrombin-III /protein-C/protein-S deficiencies, factor-V-Leiden, prothrombin mutation, paroxysmal nocturnal hemoglobinuria, dysfibrinogenemia, and use of oral contraceptives, or received fibrinolysis within the past 30 days were excluded.

Following review of literature title and abstracts, 122 manuscripts were further reviewed to determine if methods and data reported ultimately met inclusion criteria for data extraction. Manuscripts were excluded due to a variety of criteria, which included: pilot studies characterizing a non-validated test, methodology data, results did not stratify for CS, or article type was not original research such as a review article. Following final review, 48 studies were included for use in the analysis (7, 9, 10, 13, 16, 18, 19, 23–48).

### Data Extraction

Data were extracted by three independent reviewers (JW, FL, DL). Inclusion and exclusion criteria were predefined (as above) and validated through discussion with another author (MF). Studies were included if there was agreement between two independent reviewers. Discrepancies were resolved by discussion among all authors of this report on a study-by-study basis.

Data extraction was stratified into three subgroups: background, laboratory, and VTE event data. The following data were extracted for both CS and controls (if used in study) patients: number of patients, mean (± standard deviation; SD) age, gender distribution, and mean (± SD) BMI. Ethnicity was not reported in most studies. For CS patients, additional data included: source of hypercortisolism (ACTH-dependent or independent) and mean 24-h urinary free cortisol (UFC; μg/24-h) if available. Preoperative laboratory data was used for patients with a confirmed diagnosis of CS. VTE event data included the number of patients with the following outcomes for both CS and control patients: development of VTE, postoperative VTE, pulmonary embolism (PE), and VTE on prophylactic anticoagulation.

The following laboratory data was retrospectively collected: protein-S antigen, protein-C antigen, von Willebrand factor (IU/dL), plasminogen activator inhibitor-1 (IU/mL), % prothrombin time, activated partial thromboplastin time (seconds), fibrinogen (mg/dL), homocysteine (μmoL/L), % antithrombin-III, and factor VIII (IU/dL).

### Study Controls

For a control group to be acceptable given the heterogeneity of different criteria among included studies, studies were at minimum required to specify if they enrolled healthy or patients with other adrenal or pituitary diseases, and age-matched controls. However, no controls from included studies developed VTE. Therefore, we referenced the literature to determine the baseline risk of VTE in the general population to act as the control group in our analysis. A large review (49) among people of European ancestry estimated annual incidence of VTE from 104 to 183 per 100,000 person years. Using this population data as a control, we calculated the odds ratio (OR) using the higher estimate of 183 cases of VTE per 100,000 person years in the general population.

Subsequently, to calculate the odds of VTE for CS patients in the perioperative setting, we selected the control group consisting of orthopedic repair for hip or knee fractures in patients who received prophylactic anticoagulation with low molecular weight heparin. This patient population currently is indicated for perioperative anticoagulation due to a well-documented risk of VTE. Comparing the odds in both patient populations head-to-head allowed us to compare the odds of known high-risk population (orthopedic fracture) to that of an unknown population (CS). Since a minority, 5.89% (385/6,537) of the CS patients included in our analysis received anticoagulation in the perioperative period, we opted for a control group also treated with anticoagulation (incidence of VTE 1.5%; 36/2,420) (50). Conversely, we used the risk of pulmonary embolism in patients undergoing surgery for femur fracture without thromboprophylaxis in order to calculate the OR for the remaining 94.11% percent of our CS cohort undergoing surgery without anticoagulation: the estimated risk of VTE in the control arm was 14% (21/150) (51).

### Statistical Analysis

Laboratory results from all studies were pooled for analysis. Descriptive statistics using mean ± SD, and 95% confidence intervals were calculated for the pooled data. Analysis of pooled data was conducted via two-sided *t*-tests with assumed equal variance, comparing diagnosis of CS vs. control patients. We performed simple linear regression for the statistically significant laboratory values and percentage of CS patients who developed VTE.

We calculated the OR of VTE in CS patients compared to controls using outcomes data from the included studies. For patients undergoing surgery, data on the time of VTE diagnosis relative to operation was collected and post-operative period was defined as within 30 days after surgery.

Heterogeneity among studies was assessed by calculating an *I*^2^ statistic, as well as assessing the visual variability between studies by funnel plot. All analyses were conducted with STATA 15.1 statistical software (StataCorp. 2017. Stata Statistical Software: Release 15. College Station, TX: StataCorp LLC).

## Results

### Background Analysis

Characteristics of the 48 included studies are shown in Tables 2–4. Eleven studies contributed data to both outcome of VTE analysis and outcome laboratory data analysis. Thirty-five studies were retrospective in design, compared to 14 prospective studies. Twenty-one studies were cross-sectional analyses and 25 were case series. Of the remaining 2 studies, one was a survey, and one a cohort study.

**Table 2:** Demographic Data for VTE Outcome.

	**Author, Year**	**Study type**	**CS patients (*n*)**	**Causes of CS (*n*)**	**Age (years)**	**% Female**	**Controls (*n*)**
1	Bolland (35)	Survey	234	Pituitary = 188 Adrenal = 46	39	76	n/a
2	Boscaro (31)	Case series	232	Pituitary = 151 Adrenal = 67 Ectopic = 14	38.9	79	80
3	Boscaro (31)	Case series	75	Pituitary = 33 Adrenal = 27 Ectopic = 15	36.6	79	n/a
4	Chapius (27)		82	Pituitary = 78 Adrenal = 3 Ectopic = 1	41	74	n/a
5	Guarnotta (52)	Case series	192	Pituitary = 138 Adrenal = 54	43	80	n/a
6	Lambert (38)	Case series	346	Pituitary = 310 Adrenal = 36	39.9	77	n/a
7	Patil (53)	Registry	3,525	Pituitary = 3,525	n/a	82	n/a
8	Small (23)	Case series	43	Adrenal = 43	40	86	n/a
9	Stuijver (13)	Case series	473	Pituitary = 360 Adrenal = 113	42.3	76.6	n/a
10	Sudhakar (33)	Case control	22	Pituitary = 22	n/a	n/a	n/a
11	Terzolo (41)	Case control	75	Pituitary = 50 Adrenal = 19 Ectopic = 6	54.2	77	60
12	Welbourn (24)	Case series	79	Adrenal = 79	37.2	67	n/a
13	Zilio (54)	Case series	84	Pituitary = 84	42	80	n/a
14	Zilio (47)	Case series	176	Pituitary = 142 Adrenal = 24 Ectopic = 9	48	81	n/a

**Table 3:** Demographic Data for Perioperative VTE Outcome.

	**Author, Year**	**Study type**	**CS patients (*n*)**	**Surgery (*n*)**	**Age (years)**	**% Female**	**Controls (*n*)**
1	Barbot (42)	Case series	34	TSS = 34	43.1	88	n/a
	Barbot (42)	Case series	44	TSS = 44	43.3	73	n/a
2	Barzaghi (34)	Case control	288	TSS = 288	n/a	n/a	n/a
3	Boscaro (31)	Case series	232	TSS = 151			
4	Boscaro (31)	Case series	75	TSS = 46 Adrenal = 27			
5	Chapuis (27)	Case Series	82	Adrenal = 82	41	74	n/a
6	Koutroumpi (19)	Case series	57	TSS = 57	41.4	78	n/a
7	Lambert (38)	Case series	125	TSS = 125	39.9	77	n/a
8	Manetti (55)	Case control	40	TSS = 36 Adrenal = 4	46	80	40
9	McCance (26)	Case series	26	Adrenal = 26	46	77	n/a
10	Rees (56)	Case series	54	TSS = 54	41.3	78	n/a
11	Semple (28)	Case series	105	Adrenal = 105	38.5	79	n/a
12	Siren (30)	Case series	4	Adrenal = 4	n/a	n/a	n/a
13	Sudhakar (33)	Case series	22	TSS = 22	n/a	n/a	n/a
14	Turrentine (46)	Case series	36	Adrenal = 36	n/a	n/a	n/a
15	van Heerden (57)	Case series	91	Adrenal = 91	45	81	n/a
16	Zilio (47)	Case series	6	TSS = 6	48	81	n/a

**Table 4:** Demographic Data for Laboratory Outcome.

**1**	**Author, Year**	**Study type**	**CS patients (*n*)**	**Tissue origin of CS (*n*)**	**Age (years)**	**% Female**	**Controls (*n*)**
2	Barbot (42)	Case series	44	Pituitary = 44	43.2	77	n/a
3	Birdwell (58)	Case series	54	Pituitary = 52 Adrenal = 2	15.1	61	18
4	Boscaro (31)	Case-control	232	Pituitary = 151 Adrenal = 67 Ectopic = 5	38.9	79	80
5	Casonato (8)	Case series	20	Pituitary = 11 Adrenal = 7 Ectopic = 2	n/a	70	n/a
6	Chopra (36)	Case control	50	Pituitary = 22 Adrenal = 28	21.5	n/a	50
7	Colao (59)	Case control	15	Pituitary = 15	25	80	30
8	Erem (60)	Case control	24	Pituitary = 13 Adrenal = 11	41	79	24
9	Fatti (9)	Case control	29	Pituitary = 26 Adrenal = 3	39	82	20
10	Guarnotta (52)	Case series	192	Pituitary = 138 Adrenal = 54	43	80.2	n/a
11	Ikkala (16)	Case series	12	Pituitary = 7 Adrenal = 4 Ectopic = 1	28	92	n/a
12	Kastelan (18)	Case control	33	Pituitary = 25 Adrenal = 8	45.4	76	31
13	Kastelan (61)	Case control	18	Pituitary = 15 Adrenal = 3	38.6	89	18
14	Koutroumpi (19)	Case series	58	Pituitary = 43 Adrenal = 13 Ectopic = 2	41.4	78	n/a
15	Koutroumpi (40)	Case control	33	Pituitary = 24 Adrenal = 5 Ectopic^*^ = 4	52	73	30
16	Manetti (55)	Case control	40	Pituitary = 36 Adrenal = 4	46	80	40
17	Patrassi (25)	Case control	30	Pituitary = 19 Adrenal = 11	38.9	83	30
18	Giraldi (62)	Case control	12	Pituitary = 12	47.6	83	10
19	Prazny (63)	Case control	29	Pituitary = 22 Adrenal = 6 Ectopic^*^ = 1	47	72	16
20	Swiatkowska-Stodulska (64)	Case control	35	Adrenal = 35	56	71	33
21	Swiatkowska-Stodulska (37)	Case control	35	Adrenal = 35	56	71	33
22	Swiatkowska-Stodulska (45)	Case control	30	Pituitary = 12 Adrenal = 18	56.5	80	30
23	Tauchmanova (32)	Case control	28	Adrenal = 28	56	68	100
24	Terzolo (17)	Case control	41	Pituitary = 25 Adrenal = 10 Ectopic^*^ = 6	48.2	66	105
25	Tripodi (48)	Case control	48	Pituitary = 48	45	75	48
26	Zilio (47)	Case series	176	Pituitary = 142 Adrenal = 25 Ectopic^*^ = 9	48	81	n/a

In total, pooled studies represented 7,142 patients with endogenous hypercortisolism with mean follow-up of 38.89 ± 25.49 months. The underlying cause of hypercortisolism was CD in 6,174, adrenal origin in 888, ectopic CS in 80. Mean age of patients was 42.1 years of age (range 15.1–56.5 years), and patients were more likely to be female (76.2% ± 8.7), with mean BMI of 29.3 ± 1.7 kg/m^2^. Baseline UFC for patients with CS was 1061.9 ± 499.2 nmol/24 h which corresponds to 7 to 8 times upper limit of normal (ULN).

### Laboratory Analysis

Laboratory coagulation metrics are shown in Table 5. The pooled mean values for CS patients were compared to values for controls. Of those evaluated, vWF, protein-C antigen, protein-S antigen, activated partial thromboplastin time (aPTT), fibrinogen, and factor VIII had statistically significant differences between CS and control patients. Some prothrombotic parameters were noted: mean vWF in CS was higher: 180.03 ± 64.20 IU/dL (95% CI 145.81–214.24) vs. 112.53 ± 20.06 IU/dL (95% CI 98.18–126.88) in controls (*p* < 0.01), mean fibrinogen was also elevated in CS 367.85 ± 63.90 (95% CI 333.80–402.00) vs. 310.43 ± 69.04 (95% CI 266.57–354.30) in controls (*p* < 0.05), mean factor VIII in CS was higher: 168.94 ± 6.39 (95% CI 155.13–182.75) vs. 123.42 ± 72.43 (95% CI 71.61–175.24) in controls (*p* < 0.05) and mean aPTT in CS was lower: 26.91 ± 0.56 (95% CI 25.81–28.0) vs. 30.65 ± 2.95 (95% CI 28.78–32.52) in controls (*p* < 0.001). On the other hand, other antithrombotic parameters were observed in CS: Mean protein-C antigen in CS was increased: 142.18 ± 8.59 (95% CI 136.72–147.63) vs. 108.74 ± 13.69 (95%CI 97.29–120.18) in controls (*p* < 0.00001). Mean protein-S antigen in CS was also elevated: 112.25 ± 20.90 (95% CI 98.21–126.28) vs. 85.74 ± 17.65 (95% CI 69.42–102.06) in controls (*p* < 0.05).

**Table 5:** Laboratory Data in Patients with CS.

	**# CS studies**	**Mean value (SD)**	**# control studies**	**Mean value**	***p*-value**
**PROTHROMBOTIC FACTORS**					
vWF (IU/dL)	16	180.03 (64.20)	10	112.53 (20.06)	0.004
PAI-1 (IU/mL)	13	22.99 (23.94)	10	23.35 (29.65)	0.9750
Prothrombin time	10	104.49 (6.78)	5	94.49 (6.79)	0.02
aPTT (sec)	19	26.91 (2.28)	12	30.65 (2.95)	0.0004
Fibrinogen (mg/dL)	16	367.85 (63.90)	12	310.43 (69.04)	0.03
Homocysteine (μmol/L)	2	15.0 (3.96)	2	11.4 (1.27)	0.35
AT-III (%)	12	109.26 (26.09)	8	88.98 (28.45)	0.12
Factor-VIII (IU/dL)	14	168.94 (23.92)	10	123.42 (72.43)	0.04
**ANTITHROMBOTIC FACTORS**					
Protein-S Ag	11	112.25 (20.89)	7	85.74 (17.65)	0.01
Protein-C Ag	12	142.18 (8.59)	8	108.74 (13.69)	< 0.0000

Simple linear regression revealed no linear relationship between laboratory metrics and number of thrombotic events (Table 6). We additionally performed regression analysis that demonstrated no coagulation related labs were linearly associated with the severity of CS as evidenced by UFC elevation.

**Table 6:** Laboratory variables tested in associated with development of VTE following simple linear regression.

**Laboratory**	**Number of studies (*n*)**	***P*-value**	***R*^**2**^**
Protein-S	6	0.710	0.002
Protein-C	6	0.320	0.052
vWF	6	0.490	0.126
PAI-1	6	0.760	0.026
Prothrombin time	6	0.154	0.295
aPTT	8	0.651	0.036
Fibrinogen	5	0.240	0.415
AT-III	6	0.868	0.008
Factor-VIII	5	0.365	0.274

### Odds of VTE in Patients With CS Compared to General Population

**Figure 2:**
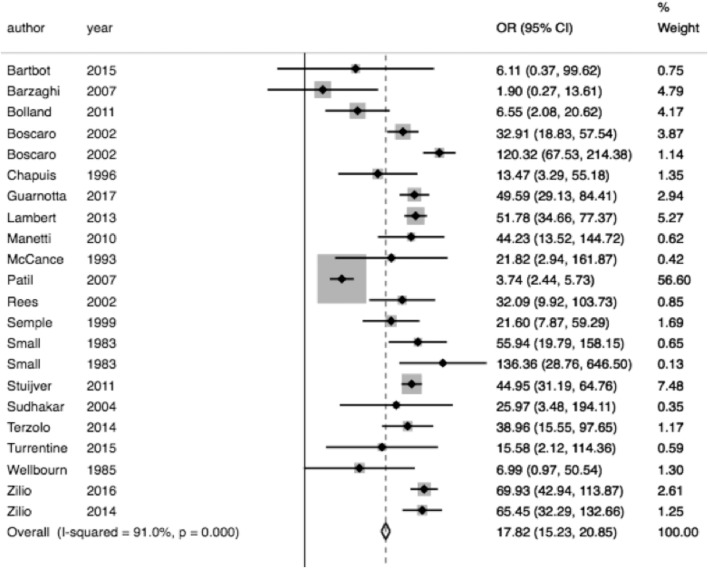
Increase odds of VTE in CS. Total OR 17.82 (95% CI 15.24–20.85) of VTE in CS compared to general population when unadjusting for VTE within 30 days of an operation. However, the interpretability of this analysis is limited by the degree of heterogeneity of odds ratios between studies included, as indicated by the *I*^2^ statistic of 91.0%.

**Figure 3:**
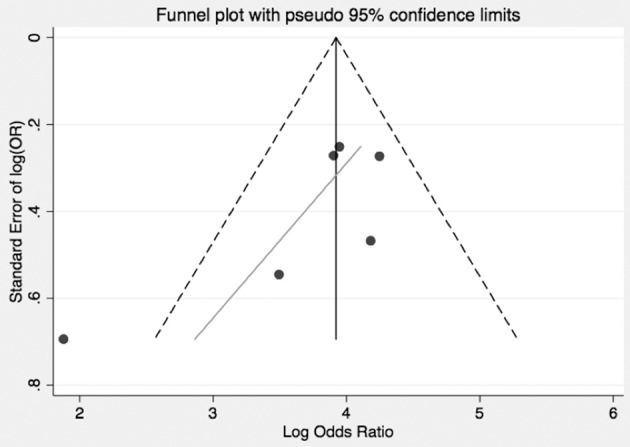
Bias and overestimation of risk impact interpretation of risk of VTE in CS from literature. Funnel plot visually representing the heterogeneity among trials reporting odds of VTE in CS. As shown, included studies are asymmetrically distributed relative to the point estimate. This suggests that pooled results are influenced both by bias and overestimate of risk in the CS population.

In pooled analysis, 3.21% (210/6,537) of CS patients experienced VTE. 62 of these events were due to pulmonary embolism and 48 deaths were attributed to VTE. The determined odds ratio is 17.8 when unadjusted for perioperative risk (95%CI 15.24–20.85, *p* < 0.00001; Figure 2). Confidence intervals were wide and displayed non-uniform overlap. Calculated I-squared statistic was highly elevated at 91%, suggesting a large degree of heterogeneity among reported odds of VTE in patients with CS. Follow-up funnel plot in assessment of bias demonstrated the cumulative literature reporting cases of VTE in patients with underlying CS is likely subject to bias and is additionally influenced by overestimation of risk (Figure 3). Other inherent risk factors for VTE were included in pooled analysis to evaluate for prognostic association for the following parameters: age, sex, active smoker, undergoing surgery (transsphenoidal surgery-TSS or bilateral adrenalectomy), and history of diabetes mellitus, when available. Simple linear regression did not indicate a significant association between any of these and the outcome of VTE (Table 7).

**Table 7:** Additional Risk factors tested in association with outcome of VTE in pooled CS cohort following simple linear regression.

**Risk factor**	**Total no. of CS patients (*n*)**	***P*-value**	***R*^**2**^**
Age	7,142	0.843	0.002
Sex	7,142	0.709	0.006
Smoker	102	0.555	0.074
Transsphenoidal surgery	721	0.629	0.088
Bilateral adrenalectomy	18	0.901	0.006
Diabetes Mellitus	604	0.686	0.017

### Odds of Perioperative VTE in Patients With CS Compared to Patients Undergoing Orthopedic Surgery

**Figure 4:**
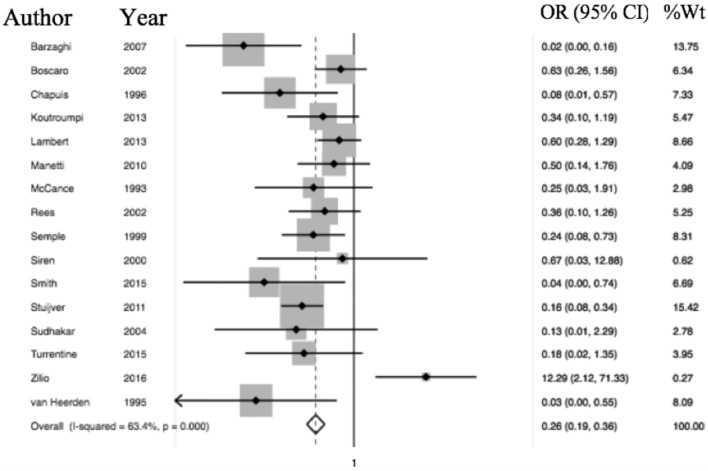
Odds of VTE in CS without anticoagulation significantly less than hip fracture surgery without anticoagulation. Total OR 0.26 (95% CI 0.19–0.36) of postop (within 30 days of surgery) VTE in CS without anticoagulation compared to odds of postoperative VTE in cases of hip fracture repair without anticoagulation. This suggests the odds of VTE in CS patients in the perioperative period for those undergoing adrenalectomy or TSS are 84% less than that of patients undergoing surgery for hip fracture.

**Figure 5:**
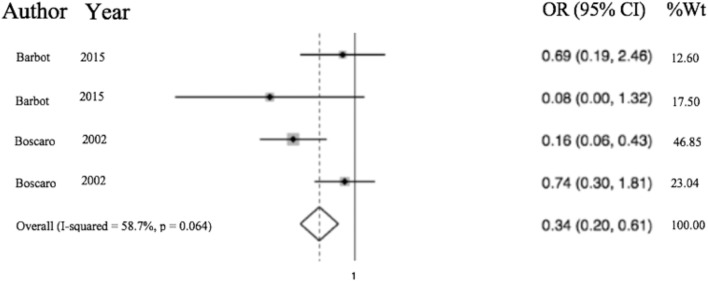
Odds of VTE in CS on anticoagulation significantly less than hip fracture surgery with anticoagulation. Total OR 0.34 (95% CI 0.20–0.61) of postop (within 30 days of surgery) VTE in CS with anticoagulation compared to odds of postoperative VTE in cases of hip fracture repair with anticoagulation. This suggests the odds of VTE in CS patients in the perioperative period for those undergoing adrenalectomy or TSS are 66% less than that of patients undergoing surgery for hip fracture.

Of the 6,537 cases included, a total of 2,083 operative cases were patients undergoing surgery, either transphenoidal surgery (TSS) or adrenalectomy for CS was undertaken. A total of 1,476 TSS cases were included compared to 607 adrenalectomies for from 16 studies. 4.75% (99/2,083) of patients had a VTE event within 30 days of surgery. The determined odds ratio for perioperative VTE in CS vs. hip fracture repair without anticoagulation is 0.26 (95% CI 0.19–0.136, *p* < 0.00001; Figure 4). Confidence intervals were wide and displayed non-uniform overlap. Calculated I-squared statistic of 63.4% indicates the greater body of literature reporting VTE events in CS patients related to surgery has less heterogeneity when accounting for the CS population as a whole, but sizable heterogeneity remains. The determined odds ratio for perioperative VTE in CS vs. hip fracture repair with anticoagulation is 0.34 (95% CI 0.20–0.61, *p* < 0.00001; Figure 5). Confidence intervals were wide and displayed non-uniform overlap. Calculated I-squared statistic of 58.7% indicates the greater body of literature reporting VTE events in CS patients related to surgery has less heterogeneity when accounting for the CS population as a whole, but sizable heterogeneity remains. In assessing for the potential influence of bias, funnel plot demonstrates moderate asymmetrical distribution of included studies (Figure 3). This suggests our pooled analysis comparing the odds of VTE in CS patients vs. the control data from trials reporting the risk of VTE in hip surgery is less subject to bias and may accurately represent a reasonable estimation of the perioperative risk of VTE in CS patients.

Only two studies reported use of thromboprophylaxis in CS and their results are presented in Table 8. However, this data is insufficient to support universal use of anticoagulation in CS undergoing intervention.

**Table 8:** Studies reporting perioperative thromboprophylaxis (*n* = 2).

**Author, year**	**Patients (*n*)**	**Observation period**	**Anticoagulation**	**Postop VTE; % (*n*)**	**Type of VTE**
Barbot (42)	34	2001–2005	Enoxaparin 4,000 UI/day or equivalent × 14 days with universal GC coverage	8.8 (3/34)	1 DVT only 1 non-fatal PE 1 fatal PE
	44	2006–2012	Enoxaparin 4,000 UI/day, or equivalent × 30 days. Elastic compression stockings. Early mobilization. GC initiated if low cortisol.	0 (0/44)	n/a
Boscaro (31)	75	1972–1981	No anticoagulation	6.6 (5/75)	4 DVT only 1 fatal PE
	232	1982–2000	Heparin 15,000–22,500 U/24 h × 22 days followed by warfarin	3.0 (7/232)	3 DVT only 1 non-fatal PE 3 fatal PE

## Discussion

An association between CS and a hypercoagulable state has been previously suggested (11, 13, 65), however, prevalence of VTE in patients with endogenous CS remains unclear. Furthermore, the role of anticoagulation is unknown. To shed light on this we undertook a systematic review of published studies to conduct a meta-analysis regarding the reported odds of VTE in CS patients compared to the general population. Subsequently, in attempt to clarify the potential role of perioperative anticoagulation to avert this risk, we compared the odds of VTE in CS patients undergoing surgery to those of patients undergoing hip replacement. This was done in order to identify whether the odds is reasonably similar between the two by comparing them head-to-head, where similar odds would argue a greater likelihood CS patients would benefit from anticoagulation, since thromboprophylaxis indication is well established in hip surgery patients.

We found that the odds of VTE is significantly increased in CS patients compared to the general population. The calculated odds of VTE in CS patients in this study was 17.82 vs. general population. Patients with CS in our meta-analysis were most often females with a mean age of 42 years, while expected rate of VTE in young women is much lower than in general population, 27 per 100,000 person-years (66), which would have resulted in much higher OR of VTE comparing to the specific CS subpopulation. No linear relationship was found correlating the severity of endogenous hypercortisolism with the number of thrombotic events or laboratory metrics coagulation. Other studies have confirmed that clinical manifestations of CS do not always correlate with the severity of hypercortisolism (52) thus we can assume the same rationale may be true for hypercoagulation and VTE.

The non-operative VTE risk in hospitalized patients is highly variable and depends on many individual factors including age >70 years malignancy, acute infection, etc. Pooled risks of inpatient-population average 0.8% of DVT and 0.4% of pulmonary embolism, but in a high- risk population, risk may increase up to 11% (67). To date, no risk assessment scales nor models have included Cushing/hypercortisolism (68–71). A key question for clinicians is whether this risk should be mitigated by treatment with anticoagulation. On the other hand, risks to anticoagulation include major bleeding, which has been reported to be between 2.8 and 6 per 100 person years (72). The risk-benefit analysis in deciding to treat with anticoagulation however necessitates indicated patients be at substantial risk of VTE in order to justify acceptable risk of hemorrhage.

In the perioperative setting, we show that the odds of VTE in patients undergoing TSS or adrenalectomy, is substantially lower than patients undergoing orthopedic surgery for hip fracture repair. CS carries a lower risk than a procedure for which prophylactic anticoagulation is currently the standard of care. Though this analysis prevents a definitive comment on risk, we can assume that CS-associated VTE risk is intermediate between general population and orthopedic surgery. Decision of prophylactic anticoagulation in CS patients undergoing surgery must then be individualized. Furthermore, bleeding risk should also be balanced in this population, especially in patient undergoing neurological surgery.

Interestingly, multiple prothrombotic laboratory metrics (vWF, fibrinogen, factor-VIII) were increased, while others were decreased (aPTT) in CS patients and anti-thrombotic markers (protein-C and protein-S) appear paradoxically to be elevated. To our knowledge, this is the first systematic review to characterize the association of increases of both protein-C and protein-S with CS. The increase in antithrombotic protein-C and protein-S has been described and is thought to be a compensatory physiological response to higher levels of clotting factors, such as FVIII, in order to promote degradation of factor excess (40, 60, 64, 73). This compensatory response could possibly vary from patient to patient and may explain why some individuals are at higher VTE risk. The underlying mechanism for this finding is likely related to a significant increase in prothrombotic metrics such as vWF, fibrinogen, and factor-VIII. All of these factors play a role in the activation of the coagulation cascade through either the intrinsic or extrinsic pathways, see Figure 2 in Isidori et al. (22). For example, vWF mediates platelet adhesion at site of endothelial damage and stabilizes FVIII, and FVIII has been described as a risk factor for VTE formation (22, 74). A corresponding decrease in aPTT, which reflects increased tendency to form clots, further supports that enhanced coagulation cascade activation occurs in these patients. The physiological explanation to link hypercortisolism and hypercoagulability is not fully understood. In one study, level of factor VIII positively correlated with UFC levels (11, 58), (not observed in our meta-analysis). Obesity also confers a higher risk of VTE, but the risk in CS seems higher than in otherwise healthy obese patients who are at low risk (14). Inflammation may induce a hypercoagulable state, but this is counterintuitive in cases of endogenous CS, since high levels of cortisol should rather have an anti-inflammatory effect (75). Many VTE events occur postoperatively and previous studies have suggested that an abrupt decrease in cortisol after surgery can increase VTE risk (2, 39, 42). A rapid drop in cortisol level could lead to a paradoxical increase in coagulation factors by withdrawal of the anti-inflammatory effect of cortisol. However, in our meta-analysis, there was insufficient data concerning remission of CS post-surgery, and adrenal insufficiency by itself does not confer an increased risk of VTE (76). Moreover, an increase in anti-coagulation parameters has been observed post-surgery, in pediatric and adult patients (11, 13, 77). Further, VTE risk decreased, but persisted even in patients who underwent anticoagulation therapy (31).

Karamouzis et al. reported data on increase platelet aggregation measured by thromboxane B_2_ levels and increased oxidative stress in patient with CS (not measured in our meta-analysis) (76). This may result in both arterial and venous increased risk of thrombosis. A systematic review in 2009 found an increased risk of VTE in CS patients, both postoperatively (0–0.6%) and unprovoked by surgery (1.9–2.5%) (65). A difference in methods between our analyses was inclusion of case series, due to limited availability of prospective studies; we also calculated an OR as these case series studies lack the rigor to properly define risk. Thus, outcome of VTE events from our analysis is relatable, though not identical in nature. Our analysis also included 33 articles additional articles beyond the 2009 (2010–2017), which represents a significant body of literature and warrants revisiting this topic (65).

Our meta- analysis further confirms an association between CS and VTE, and changes in coagulation parameters. Clinicians and patients should be aware of this association, and CS management tailored on an individual basis. A recent Pituitary Society survey showed awareness regarding hypercoagulability in CD quadrupled in 2 years (78); routine VTE prophylaxis increased from 50 to 75% peri-operatively and doubled in patients who underwent bilateral inferior petrosal sinus sampling (BIPSS). However, prophylactic treatment for hypercoagulability was not universally administered. For centers using anticoagulation, low-molecular heparin was the preferred agent for VTE prophylaxis and most of the responders used it for approximately 2 weeks post-operatively. It is the authors' opinion that more rigorous trials are needed to identify additional VTE risk factors and possible mitigating factors.

Steps such as screening coagulation metrics in CS patients or even considering anticoagulation may be appropriate, especially in perioperative setting or around procedures such a BIPSS. However, this cannot be definitely inferred from the data we present. Such recommendations require well designed, prospective analyses. Nevertheless, screening CS patients for inherited coagulation pathologies and other additional risk factors would help determine the highest “at risk” subgroup of patients (22). Regarding timing of anticoagulation, in patients with gliomas (albeit with much higher risk of VTE), recent guidelines suggest VTE prophylaxis with low-molecular-weight heparin in brain tumor patients to be started postoperatively within 24 h to decrease risk of hemorrhage (79). A similar pattern can be potentially applicable to selected high risk VTE cases in patients with CS, but further studies are needed.

Data on medical therapy directed to lower cortisol values on VTE risk is sparse (80). In a study of 17 patients with CD on short-term triple combination medical therapy (pasireotide, cabergoline and ketoconazole), both increased production of procoagulant factors and impaired fibrinolysis, have not been shown to reverse despite biochemical remission after successful medical therapy (81). The authors found a slight decrease in antithrombin levels after 3 months of successful medical therapy (levels were normal at baseline), but no changes in fibrinogen, D-dimer levels, factor VIII activity, and protein C and S activity. Another prospective study in 21 patients treated with short-acting pasireotide confirmed the lack of effect of medical therapy of CS on changing either clotting or anticoagulant factors during therapy; no patients developed thrombotic complications during treatment (81).

## Limitations

There are inherent study limitations. Inclusion of case series and lower quality studies rules out a determination of risk *per se* in the meta-analysis, as presented. To address this we computed an OR. The OR calculations were, however, limited by the lack of controls who developed VTE, which was addressed by referencing epidemiologic data from a population of white-European descent, the majority of patients in the CS studies. Additionally, the inclusion and exclusion criteria varied between the included studies. We attempted to address this via verification that authors at a minimum specified that controls were health- and age-matched in an attempt to limit variation *a priori*. However, when determining the OR of VTE in CS, the degree of heterogeneity indicates the included studies differed greatly (Figures 4, 5). Unfortunately, factors required to evaluate this concern, such as severity of illness, methods of diagnosis for both CS and VTE were not available. The diagnosis of VTE was clinical for most studies or studies did not objectively report how VTE was diagnosed. For example, confirmatory testing for deep vein thrombosis with doppler ultrasound and venography with rule in and rule out patients under different operative characteristics of that given test (i.e., sensitivity and specificity). Since no standardized diagnosis method was used to make the diagnosis of VTE, we are left to assume each patient who had a reported VTE event per study did indeed have the appropriate diagnosis regardless of workup and did not involve systematic search for VTE, some events may have been missed and VTE prevalence underestimated. Concomitant interventions such as elastic stockings and early mobilization may not have been reported or may not have been consistent in studies over a long observation period. As previously mentioned, trial designs reviewed and included are of lower evidence quality, which lends the pooled results to the biases of such designs. However, the degree of heterogeneity is, impart, and accounted for by these biases as evidenced in the funnel plot where smaller studies are distributed asymmetrically (Figure 3). Lastly, due to the lack of prospective data on this topic, we utilized the literature to provide data on the risk of VTE in all of our control calculation. The risk data in the control group was used in order to calculate odds, resulting in data from multiple trial designs being synthesized into a single calculation in order to perform the meta-analysis.

## Conclusions

Endogenous Cushing's syndrome is associated with significantly high odds of developing VTE. Our meta-analysis, which includes case-series in addition to prospective and retrospective studies, shows that risk of VTE (both non-operative and post-operative cases) in CS is significantly increased compared with general population. Rates of VTE in CS seem lower than in patients with hip fracture postsurgery, a disease well- known to carry high risk of VTE and in which most patients require prophylactic anticoagulation. Nonetheless, a definitive determination of risk and benefits of anticoagulation in CS is needed through large, prospective controlled trials. Exact timing (at diagnosis or before and after surgery), type, doses and duration of prophylaxis medications remains to be established. It is essential that endocrinologists bear in mind the close association of CS with VTE when caring for patients and consider prophylactic intervention and/or monitoring on an individualized basis.

## Author Contributions

JW, FL, DL, and MF contributed to study conception and design, acquisition of data, and analysis and interpretation of data. JW, FL, SM, and MF drafted the manuscript. JW, FL, DL, SM, and MF critically revised and reviewed the submitted version of the manuscript. JW and FL contributed to statistical analysis. SM and MF provided administrative and technical support. MF provided overall study supervision.

### Conflict of Interest Statement

The authors declare that the research was conducted in the absence of any commercial or financial relationships that could be construed as a potential conflict of interest.
